# Interleukin-1beta and fibroblast growth factor receptor 1 cooperate to induce cyclooxygenase-2 during early mammary tumourigenesis

**DOI:** 10.1186/bcr2246

**Published:** 2009-04-24

**Authors:** Johanna R Reed, Ronald P Leon, Majken K Hall, Kathryn L Schwertfeger

**Affiliations:** 1 Microbiology, Immunology and Cancer Biology Graduate Program, University of Minnesota, 420 Delaware St. SE, Minneapolis, Minnesota 55455, USA; 2 Department of Laboratory Medicine and Pathology and Masonic Cancer Center, University of Minnesota, 420 Delaware St. SE, Minneapolis, Minnesota 55455, USA

## Abstract

**Introduction:**

Inflammation within the tumour microenvironment correlates with increased invasiveness and poor prognosis in many types of cancer, including breast cancer. We have previously demonstrated that activation of a mouse mammary tumour virus (MMTV)-driven inducible fibroblast growth factor receptor 1 (iFGFR1) transgene in mammary epithelial cells results in an inflammatory response characterised by induction of inflammatory genes in the mammary gland. Specifically, we have observed increased levels of IL-1β expression in the mammary gland following activation of iFGFR1 and have used the iFGFR1 model to elucidate the function of IL-1β in promoting iFGFR1-induced mammary lesions.

**Methods:**

To determine the functional consequences of IL-1β induction during FGFR1-induced mammary tumourigenesis, the effects of IL-1β inhibition on the formation of epithelial hyperplasias were examined using the MMTV-iFGFR1 transgenic mouse model. Further studies used a combination of the HC-11 mammary epithelial cell line that stably expresses iFGFR1 and the MMTV-iFGFR1 transgenic mice to further define the mechanisms of IL-1β function.

**Results:**

Inhibition of IL-1β activity *in vivo *resulted in reduced iFGFR1-induced epithelial proliferation and formation of hyperplastic structures. Further studies demonstrated that treatment of mammary epithelial cells with IL-1β-induced expression of cyclooxygenase (Cox)-2 both *in vitro *and *in vivo*. Finally, inhibition of Cox-2 prior to activation of iFGFR1 in the transgenic mice also resulted in decreased iFGFR1-induced formation of hyperplastic structures.

**Conclusions:**

The results from these studies indicate that targeting the inflammatory cytokine IL-1β partially inhibits iFGFR1-induced formation of early-stage mammary lesions, in part through induction of Cox-2. These findings demonstrate that activation of a growth factor receptor in mammary epithelial cells results in increased expression of inflammatory mediators, which cooperate to promote the initiation of hyperplastic lesions in the mammary gland.

## Introduction

Inflammation is a well-known risk factor for tumour development and correlates with increased invasiveness and poor prognosis in a variety of cancers [1]. It is well-established that chronic inflammation that is driven by extrinsic factors promotes several types of cancer, including gastric, hepatic and gastrointestinal cancers [1]. However, inflammation has also been correlated with the development of cancers that are not typically associated with chronic inflammatory states, such as breast cancer. There has been ongoing interest in the concept that intrinsic factors, such as activation of an oncogene within epithelial cells, induce a state of localised inflammation that subsequently promotes tumourigenesis [2].

Recent epidemiological studies support a role for anti-inflammatory drugs in the reduction of breast cancer risk [3-7]. Furthermore, inflammatory cytokines, such as IL-1β, and other mediators of inflammation have been linked to breast cancer formation and recurrence [8,9]. Cyclooxygenase (Cox)-2 is a primary downstream target of inflammatory cytokines and has been linked to proliferation, suppression of apoptosis, induction of genomic instability, resistance to treatment and angiogenesis in breast cancer [10,11]. Although Cox-2 is commonly associated with inflammatory cells, studies have also demonstrated that Cox-2 is induced by activation of oncogenes within breast cancer cells [12,13]. Therefore, Cox-2 is likely to represent an important component of intrinsically induced inflammation in breast cancer. Understanding the mechanisms by which intrinsic factors, such as oncogenes, induce inflammation is critical for successfully developing and using anti-inflammatory strategies to target breast cancer formation and recurrence.

Using an inducible mouse model of mammary tumourigenesis, we previously demonstrated that activation of an inducible fibroblast growth factor receptor-1 (iFGFR1) transgene within epithelial cells resulted in the formation of hyperplastic budding structures within 48 hours of iFGFR1 activation [14,15]. These early structures were characterised by increased proliferation and lack of formation of a proper lumen surrounded by the epithelium [15]. Longer-term activation demonstrated that iFGFR1 activation promoted a loss of myoepithelial cells, and increased angiogenesis, formation of locally invasive lesions and, ultimately, mammary tumour formation [14,15]. Further studies of this model demonstrated that iFGFR1 activation in the mammary gland induced a rapid, localised inflammatory response, characterised by recruitment of macrophages to the epithelial structures and induction of inflammatory genes by microarray analysis [14]. Activation of iFGFR1 has been shown to promote proliferation, survival, migration, invasion and epithelial-mesenchymal transition of mammary epithelial cells [16]. Furthermore, FGFR1 is amplified in 10% of human breast tumours and has been linked to a poor response to treatment in breast cancer patients [17]. However, the mechanisms by which FGFR1 activation in epithelial cells induces pro-tumourigenic effects in the microenvironment are only beginning to be understood.

Our current studies focus on the ability of iFGFR1 activation to promote an intrinsic pathway of inflammation using a transgenic mouse model. We have found that activation of iFGFR1 within mammary epithelial cells results in the expression of proinflammatory genes, such as IL-1β and Cox-2, and that these mediators are important for iFGFR1-induced early-stage tumourigenesis. These studies demonstrate that activation of an oncogenic growth factor signalling pathway within mammary epithelial cells induces a localised inflammatory response that promotes the formation of early-stage mammary lesions.

## Materials and methods

### Animals

Generation of mouse mammary tumour virus (MMTV)-iFGFR1 transgenic mice has been described previously [15] and the mice were obtained from Dr Jeff Rosen (Baylor College of Medicine, Houston, TX, USA). Animal care and procedures were approved by the Institutional Animal Care and Use Committee of the University of Minnesota and were in accordance with the procedures detailed in the Guide for Care and Use of Laboratory Animals.

### Treatment of mice

For iFGFR1 activation, six-week-old female mice were injected intraperitoneally (i.p.) with 1 mg/kg AP20187 (Ariad Pharmaceuticals, Cambridge, MA, USA) twice weekly. Mice were sacrificed at 48 hours and four weeks post-injection and mammary glands from at least three mice were analysed per time point. For the IL-1β neutralisation studies, the mice were treated as described previously [18]. Briefly, mice were injected i.p. with 4 μg/g IL-1β antibody (AB-401-NA, R&D Systems, Minneapolis, MN, USA) 24 hours prior to AP20187 treatment and in conjunction with AP20187 for an additional 48 hours. Control mice were injected with isotype control total immunoglobulin (Ig) G (R&D Systems, Minneapolis, MN, USA) for the same time frame. For inhibition of Cox-2 by celecoxib, four-week-old female MMTV-iFGFR1 transgenic mice and non-transgenic littermate control mice were fed standard mouse chow (Harlan Laboratories, Madison, WI, USA) enriched with celecoxib (obtained as 200 mg capsules) at a concentration of 1000 mg/kg (Harlan Laboratories, Madison, WI, USA). Celecoxib-treated mice and control mice, given standard mouse chow only, were fed for one week. All mice were then injected i.p. with 1 mg/kg AP20187. Mice were maintained on the celecoxib or control diets and were sacrificed 48 hours post-injection. Mammary glands from five mice per treatment group were removed for whole mount and histological analysis.

### Mammary gland whole mounts, histology and measurement of epithelial budding

Whole mounts were prepared as described previously [14]. For embedding, sectioning and immunohistochemistry, mammary glands were fixed for two hours in 4% paraformaldehyde and embedded in paraffin. For histological analysis, the glands were sectioned and stained with H&E using standard histological protocols. To quantify epithelial budding structures, six images were taken per mammary gland section at 10× magnification. Five sections were analysed per gland, each approximately 100 μm apart, to compensate for variability within the gland. In addition, only epithelial structures distal to the lymph node were included in the analyses due to the predominant localisation of the hyperplastic phenotype along ducts leading to terminal end buds. The total number of epithelial structures was counted and expressed as a percentage of structures that contain epithelial buds. At least three mice and 200 epithelial structures were analysed for each genotype and treatment. All statistical analyses were performed using the unpaired student's t-test to compare two means (GraphPad Prism, La Jolla, CA, USA).

### Immunohistochemistry

The following antibodies and dilutions were used for immunohistochemistry: rabbit polyclonal IL-1β (sc-7884), 1:100, goat polyclonal Cox-2 (sc-1747), 1:100, (Santa Cruz Biotechnology, Santa Cruz, CA, USA), mouse monoclonal phospho-histone H3 (pH3; 05-806, Millipore, Billerica, MA, USA). Immunostaining was performed either with (IL-1β, pH3) or without (Cox-2) sodium citrate antigen retrieval, as described previously [19]. pH3 and Cox-2 positive cells were counted and calculated relative to the number of total epithelial cells. At least 2000 cells from a total of three mice per treatment group were counted for each dataset. All statistical analyses were performed using the unpaired student's t-test to compare two means.

### RNA isolation and quantitative RT-PCR analysis

Transgenic and non-transgenic six-week mice were treated for 8, 16, 24 and 48 hours with AP20187, IgG isotype control and/or IL-1β blocking antibody as indicated. Mammary glands were isolated, the lymph nodes were removed and the mammary glands were ground under liquid nitrogen and lysed in 2 mls of Trizol (Invitrogen, Carlsbad, CA, USA). RNA was extracted from monolayer cells using Trizol as recommended by the manufacturer. cDNA was generated using the Quantitect Reverse Transcription kit (Qiagen, Valencia, CA, USA). One-tenth of the final reaction volume was used in quantitative SYBR (Synergy Brands) green RT-PCR reactions as described previously [20] using the Bio-Rad iQ5 system (Bio-Rad, Hercules, CA, USA). Relative quantification of the expression of each gene was calculated and normalised to averaged cyclophilin and glyceraldehyde 3-phosphate dehydrogenase (GAPDH) expression levels as indicated using the 2^-ΔΔCt ^method [21]. The following primer sequences were used: IL-1β 5'-GCAACTGTTCCTGAACTCAAC-3' and 5'-ATCTTTTGGGGTCCGTCAACT-3', Cox-2 5' TGAGCAACTATTCCAAACCAG-3' and GCACGTAGTCTTCGATCACTATC, cyclophilin 5'-TGAGCACTGGGGAGAAAGG-3' and 5'TTGCCATCCAGCCACTCAG-3', GAPDH 5'-TGACCACAGTCCATGCCATC-3' and 5'-GACGGACACATTGGGGGTAG-3'. All statistical analyses were performed using the unpaired student's t-test to compare two means.

### Cell culture and immunoblot analysis

Generation of HC-11 cells stably expressing the iFGFR1 construct was described previously [16] and the cells were obtained from Dr Jeff Rosen (Baylor College of Medicine, Houston, TX, USA). The cells were incubated in serum-free RPMI media for 16 hours prior to treatment with either the indicated amounts of recombinant IL-1β (Pierce Endogen, Rockford, IL, USA) or with 30 nM AP20187 for the indicated times. The cells were lysed in radio immuno precipitation assay buffer and protein-containing supernatants were generated by centrifugation. Equal amounts of protein were analyaed by SDS-PAGE and immunoblotting analysis was performed with the following antibodies at a dilution of 1:1000: phospho-p65 (3033), p65 (4764), Cox-2 (4842) and β-tubulin (2146) (Cell Signaling Technology, Beverly, MA, USA). Densitometry was performed using an AlphaImager 3400 (Alpha Innotech, San Leandro, CA, USA).

### Co-culture and ELISA analysis

RAW 264.7 cells (American Type Culture Collection, Manassas, VA, USA) and HC-11/R1 cells were plated at equal densities in six-well tissue culture plates and allowed to grow for 48 hours in complete HC-11/R1 media. The cells were incubated overnight in serum-free RPMI media and treated with either 30 nM AP20187 or an equal amount of ethanol as a solvent control. Following eight hours of treatment, the cells were harvested in Trizol and IL-1β expression levels were analysed as described above. Following 24 hours, conditioned medium was harvested and was analysed using an ELISA to quantify the amount of IL-1β in the media following the manufacturer's protocols (R&D Systems, Minneapolis, MN, USA). All statistical analyses were performed using the unpaired students t-test to compare two means.

### Migration assays

Migration assays were performed as described previously [16]. Briefly, HC-11/R1 cells were grown to confluence and incubated overnight in serum-free medium. A p20 pipet tip was used to make a scratch down the centre of the well and pictures were taken of the scratch prior to treatment of cells with 30 nM AP20187 and/or 5 ng/ml recombinant IL-1β. After 18 hours, pictures were taken again and the area of the gap closure was quantified using Leica LAS software (Leica Microsystems, Wetzlar, Germany). All statistical analyses were performed using the unpaired student's t-test to compare two means.

## Results

### Activation of iFGFR1 in mammary epithelial cells induces expression of the inflammatory cytokine IL-1β in the mammary gland

FGFR1 is amplified in approximately 8 to 10% of human breast cancers [22]. However, due to lack of a ligand that specifically activates FGFR1 without activating the other FGFRs, a model was developed to study the effects of FGFR1 activation specifically in mammary epithelial cells [15]. In this model, a modified, membrane-targeted FGFR1 (iFGFR1) that lacks an extracellular domain is activated by treatment of cells with a lipid-soluble dimeriser, AP20187. Upon binding, AP20187 induces homodimerisation and activation of the receptor. The effects of activating this FGFR1 construct in mammary epithelial cells in cell culture and *in vivo *using the MMTV-iFGFR1 transgenic mouse model have been described previously [14-16,23]. Specifically, iFGFR1 activation in the mammary gland results in the rapid formation of hyperplastic epithelial structures within 48 hours accompanied by an inflammatory response, characterised by rapid macrophage recruitment and increased expression of several inflammatory genes [14,15].

**Figure 1:**
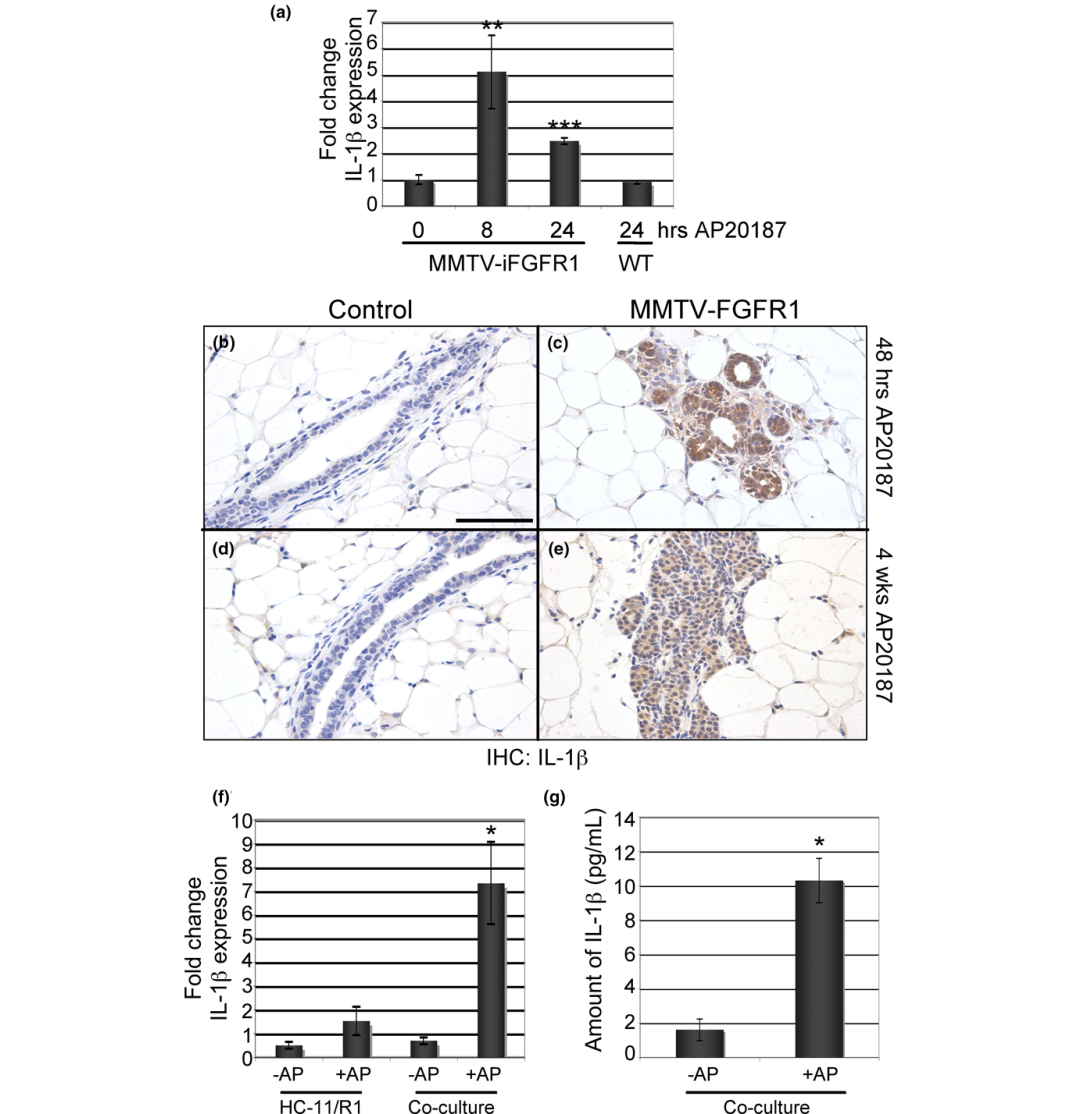
iFGFR1 activation induces IL-1β expression in the mammary gland. **(a) **Mouse mammary tumour virus (MMTV) inducible fibroblast growth factor receptor 1 (iFGFR1) transgenic mice and non-transgenic littermate controls were treated with AP20187 for 8 and/or 24 hours. Non-treated mice were used as the baseline control. Quantitative RT-PCR was performed on RNA extracted from whole mammary gland tissue and normalised to expression levels of cyclophilin. Mammary glands from three separate mice were analysed per time point. Error bars represent standard error of the mean. ** *P *< 0.01, *** *P *< 0.001. **(b to e) **Immunohistochemical analysis of mammary gland tissue sections from MMTV-iFGFR1 transgenic mice and non-transgenic littermate controls following either 48 hours or 4 weeks of AP20187 treatment. Images are representative of results from the analysis of three mice per genotype and treatment time. **(f) **Quantitative RT-PCR analysis of IL-1β expression levels (normalised to glyceraldehyde 3-phosphate dehydrogenase). Either HC-11/R1 cells or HC-11/R1-RAW264.7 co-cultures were treated with AP20187 (+AP) or solvent (-AP) for four hours and analysed for IL-1β gene expression. * *P *< 0.05. **(g) **HC-11/R1-RAW264.7 co-cultures were treated with AP20187 (+AP) or solvent (-AP) for 18 hours and conditioned media was analysed using an ELISA. * *P *< 0.05.

To identify inflammatory genes that contribute to the iFGFR1-induced inflammatory response, we used quantitative RT-PCR analysis to examine the expression of several candidate genes in the mammary gland following treatment of mice with AP20187, which activates iFGFR1. Interestingly, we found a significant induction of IL-1β, which is a critical cytokine in the inflammatory response that has also been linked to breast cancer invasiveness and recurrence [8,9]. As shown in Figure 1a, expression of IL-1β mRNA increased significantly in the mammary gland within eight hours and remained elevated following 24 hours of iFGFR1 activation. Treatment of non-transgenic littermates with AP20187 did not induce a similar increase in IL-1β expression, demonstrating that this response was not a general inflammatory reaction to either the AP20187 dimeriser or the solvent used for the injections (Figure 1a). In addition to gene expression levels, immunohistochemical analysis of mammary gland sections demonstrated an increase in IL-1β protein expression following 48 hours of iFGFR1 activation in comparison to non-transgenic littermates treated with AP20187 (Figures 1b,c). Analysis of a four-week time-point revealed sustained expression of IL-1β associated with iFGFR1-induced hyperplastic lesions (Figures 1d,e). These results demonstrate that activation of iFGFR1 in mammary epithelial cells results in expression of the key inflammatory mediator, IL-1β, in the mammary gland.

Because IL-1β is secreted, the cellular source of the IL-1β in the mammary gland is difficult to discern using immunohistochemistry. Therefore, we utilised *in vitro *assays to determine whether iFGFR1 directly induces expression of IL-1β in mammary epithelial cells. For these studies, we used a derivative of the HC-11 cell line, which is a mouse mammary epithelial cell line derived from a mid-pregnant Balb/c mammary gland [24]. To study the consequences of iFGFR1 activation in these cells, clones of HC-11 cells were previously generated that stably express iFGFR1 (HC-11/R1) [16]. Published studies have demonstrated that activation of iFGFR1 in these cells promotes proliferation, survival, migration and invasion [16,23]. Therefore, these cells represent a relevant *in vitro *model with which to examine iFGFR1-mediated mechanisms of mammary tumourigenesis.

To determine the effects of iFGFR1 activation on IL-1β expression, the cells were treated with 30 nM AP20187 and IL-1β expression was analysed using both quantitative RT-PCR and ELISA assays. Although we detected a small increase in IL-1β mRNA expression (Figure 1f), this induction was not statistically significant. Furthermore, no IL-1β protein was detected in conditioned media using an ELISA assay (data not shown). Therefore, we hypothesised that activation of iFGFR1-induced expression of IL-1β requires the presence of other cell types. We had previously observed a rapid accumulation of macrophages around the hyperplastic epithelial structures following iFGFR1 activation in the mammary gland [14]. Furthermore, macrophages are known to secrete high levels of IL-1β in response to inflammatory stimuli [25]. Therefore, we analysed IL-1β expression levels following activation of iFGFR1 in epithelial cell/macrophage co-cultures. Interestingly, significant increases in both IL-1β mRNA and protein were detected in the co-cultures (Figures 1f,g). Similar results were found when the HC-11/R1 cells were co-cultured with mouse bone marrow-derived macrophages (data not shown). These results suggest that interactions between epithelial cells and macrophages may be required to induce IL-1β following iFGFR1 activation in the mammary epithelial cells *in vitro*.

### IL-1β promotes the formation of iFGFR1-induced hyperplasias *in vivo*

**Figure 2:**
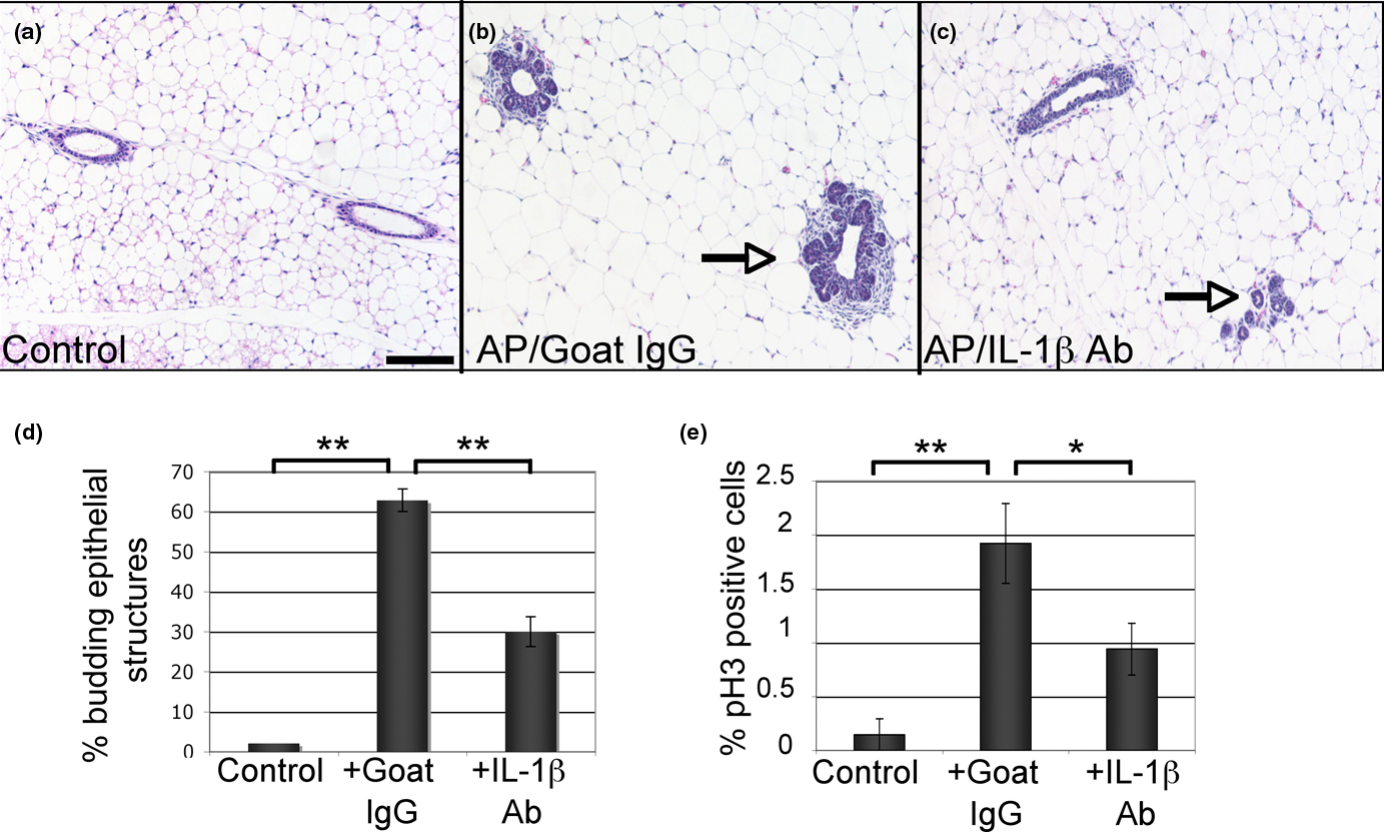
Analysis of mammary glands from iFGFR1 transgenic mice following activation of iFGFR1 and treatment with an IL-1β neutralising antibody. H&E-stained sections from **(a) **non-treated control mice, **(b) **transgenic mice treated with AP20187 and goat IgG, and **(c) **transgenic mice treated with AP20187 and IL-1β blocking antibody (Ab). Arrows indicate epithelial budding structures. Scale bar = 100 μM. **(d) **Budding structures were quantified by counting epithelial structures in H&E-stained sections (three mice per treatment group). Decreased epithelial budding was observed following treatment with AP20187 and the IL-1β antibody compared with treatment with AP20187 and IgG. ** *P *< 0.01 **(e) **Sections were immunostained with an antibody to phospho-histone H3 (pH3), a marker of mitosis, and the percentage of pH3 positive epithelial cells was determined. Decreased numbers of pH3 were observed following treatment with AP20187 and the IL-1β antibody compared with treatment with AP20187 and IgG. * *P *< 0.05, ** *P *< 0.01. iFGFR1 = inducible fibroblast growth factor receptor 1.

Based on the increased expression of IL-1β following FGFR1 activation and the link between IL-1β and breast cancer [8], we hypothesised that IL-1β may be an important factor in the formation of iFGFR1-induced proliferative lesions. To examine this hypothesis, we used a systemic IL-1β neutralisation strategy similar to that described previously [18]. For these experiments, either an IL-1β neutralising antibody or an equivalent amount of an isotype control goat IgG antibody was administered i.p. to six-week-old female transgenic mice 24 hours prior to iFGFR1 activation. The mice were then treated with AP20187 to activate iFGFR1 in conjunction with daily treatments of either IL-1β blocking antibody or an isotype control goat IgG for 48 hours. Following the treatments, the mammary glands were analysed by whole mount analysis and sections were analysed for the percentage of budding epithelial structures, proliferation and macrophage recruitment. Analysis of whole mounts revealed that IL-1β inhibition led to an overall decrease in hyperplastic budding, particularly associated with the terminal-end buds and the subtending ducts (see Additional data file 1). Further studies were performed to validate these alterations in histological sections. We found that although inhibition of IL-1β did not completely abolish hyperplastic budding of the epithelium, there was a significant decrease in the total number of budding structures present within mammary glands from mice that had been treated with the IL-1β blocking antibody (Figures 2a to 2d). Furthermore, there was a corresponding decrease in the percentage of proliferating epithelial cells as measured by quantification of pH3 immunofluorescence (Figure 2e). Interestingly, analysis of macrophage recruitment revealed that inhibition of IL-1β activity did not affect iFGFR1-induced recruitment of macrophages to the epithelium (data not shown). Together, these studies suggest that although IL-1β may not be critical for the recruitment of macrophages observed in this model, IL-1β activity contributes to epithelial proliferation during the formation of the iFGFR1-induced lateral budding phenotype.

### IL-1β induces activation of NFκB in HC-11/R1 mammary epithelial cells

**Figure 3:**
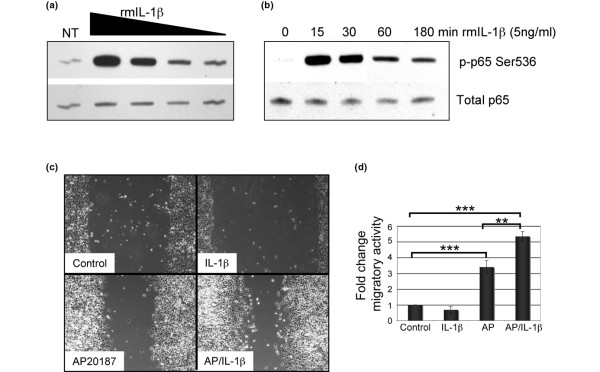
IL-1β induces NFκB activation and cooperates with iFGFR1 to promote migration in HC-11/R1 cells. **(a) **HC-11/R1 cells were incubated in serum-free medium overnight and then stimulated with the following concentration of recombinant IL-1β for 15 minutes: 50, 5, 0.5 and 0.05 ng/ml IL-1β. Immunoblot analysis was performed to detect levels of phospho-p65 (upper panel) and total p65 (lower panel) as a loading control. **(b) **HC-11/R1 cells were treated with 5 ng/ml recombinant murine (rm) IL-1β for the indicated times and immunoblot analysis was performed as described in a. **(c, d) **At confluency, HC-11/R1 cells were serum starved for 24 hours, scratched using a pipet tip and allowed to recover for 18 hours in the presence of IL-1β, AP20187 or both. Pictures were taken immediately after the scratch and 18 hours later and were used to determine the extent of migration **(d) **measuring the changes in area between the scratch surfaces. Error bars represent standard error of the mean. ** *P *< 0.01, *** *P *< 0.001. iFGFR1 = inducible fibroblast growth factor receptor 1; NF = nuclear factor.

Based on the results from the *in vivo *studies, we hypothesised that IL-1β acts on the mammary epithelial cells to contribute to the formation of iFGFR1-induced hyperplastic lesions. Therefore, initial studies were performed to ascertain the ability of mammary epithelial cells to respond to IL-1β stimulation by examining activation of downstream signalling pathways using the HC-11/R1 cell line described previously. Initial studies using both quantitative RT-PCR and immunoblot analysis demonstrated that the HC-11/R1 cells express the IL-1 receptor (IL-1R) (data not shown). Further studies were performed using both dose-response and time-course analyses to examine the activation of nuclear factor (NF) κB, which is a key downstream target of IL-1β in other cell types [26]. Treatment of HC-11/R1 cells with recombinant murine (rm) IL-1β resulted in a rapid induction of phosphorylation of the p65 subunit of NFκB within 15 minutes of treatment as shown by immunoblot analysis (Figures 3a,b). Based on the observation that 5 ng/ml of rmIL-1β was within the linear range of response (Figure 3a), this concentration was used for the remainder of the studies. These results demonstrate that the HC-11/R1 mammary epithelial cell line responds to rmIL-1β treatment by activating a well-defined downstream signalling pathway.

### IL-1β and iFGFR1 cooperate to promote migration of HC-11/R1 cells

Previous studies have demonstrated that treatment of breast cancer cells in culture with IL-1β promotes cell proliferation and migration [27,28]. Therefore, we examined the ability of IL-1β to promote these properties in non-transformed mammary epithelial cells using 3-[4,5-dimethylthiazol-2-yl]-2,5-diphenyl tetrazolium bromide and scratch wounding assays, respectively. In contrast to studies of breast cancer cells, treatment of HC-11/R1 cells with rmIL-1β alone did not promote either proliferation (data not shown) or migration (Figures 3c,d). Because IL-1β can promote these properties in breast cancer cells, we hypothesised that IL-1β may act cooperatively with another oncogenic stimulus to promote tumourigenic changes. Therefore, we stimulated HC-11/R1 cells with AP20187, which activates iFGFR1, and rmIL-1β either alone or in combination. Addition of IL-1β to the media did not significantly affect iFGFR1-induced proliferation of the HC-11/R1 cells (data not shown). However, addition of both AP20187 and rmIL-1β to the media promoted a significant increase in migration in comparison with iFGFR1 activation alone (Figures 3c,d). These studies suggest that although treatment of non-transformed mammary epithelial cells with IL-1β alone does not promote the acquisition of tumourigenic properties, IL-1β may act cooperatively with other oncogenic stimuli to promote these properties during tumour formation.

### IL-1β and iFGFR1 induce expression of Cox-2, which promotes migration of HC-11/R1 cells *in vitro*

**Figure 4:**
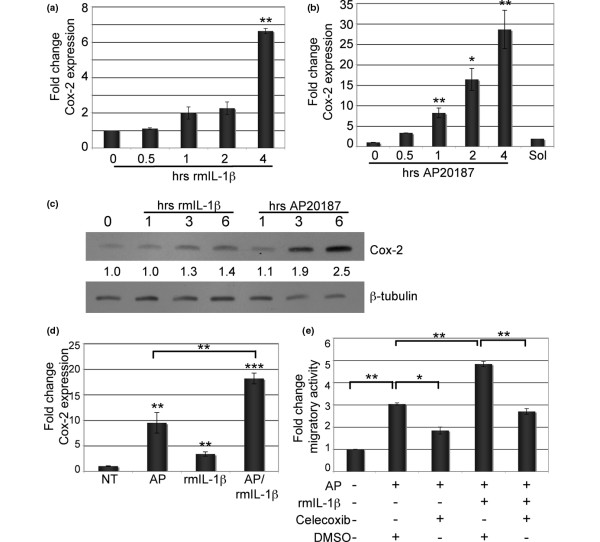
IL-1β and iFGFR1 induce expression of Cox-2 in HC-11/R1 cells. **(a) **HC-11/R1 cells were treated with 5 ng/ml rmIL-1β for the indicated times. Quantitative RT-PCR was used to analyse IL-1β mRNA expression (normalised to levels of cyclophilin and glyceraldehyde 3-phosphate dehydrogenase (GAPDH)). Error bars represent standard error of the mean. ** *P *< 0.01. **(b) **HC-11/R1 cells were treated with either 30 nM AP20187 or ethanol (Sol) for the indicated times and cyclooxygenase (Cox) 2 mRNA expression was analysed and normalised as described in a. * *P *< 0.05, ** *P *< 0.01. **(c) **HC-11/R1 cells were stimulated with either 5 ng/ml recombinant murine (rm) IL-1β or 30 nM AP20187 for the indicated times. Immunoblot analysis was performed using antibodies specific for Cox-2 and β-tubulin as a loading control. Densitometry was performed to analyse Cox-2 expression levels relative to β-tubulin. **(d) **HC-11/R1 cells were treated with 30 nM AP20187 and 5 ng/ml rmIL-1β for one hour. Quantitative RT-PCR analysis was performed to examine levels of Cox-2 expression, which were normalised to levels of cyclophilin and GAPDH. Error bars represent standard error of the mean. ** *P *< 0.01, *** *P *< 0.001. **(e) **Migration assays were performed as described in Figure 3. Cells were treated with 30 nM AP20187 and/or 5 ng/ml IL-1β in the presence of either celecoxib or dimethyl sulfoxide (DMSO) as a solvent control as indicated. Error bars represent standard error of the mean. * *P *< 0.05, ** *P *< 0.01. iFGFR1 = inducible fibroblast growth factor receptor 1.

To identify downstream targets of IL-1β in mammary epithelial cells, we asked whether IL-1β could induce expression of Cox-2, a known IL-1β target [26], in the HC-11/R1 cells. For these studies, HC-11/R1 cells were treated with rmIL-1β and Cox-2 expression was evaluated by quantitative RT-PCR and immunoblot analysis. Quantitative RT-PCR analysis demonstrated a modest induction of both Cox-2 mRNA and protein following four and six hours of IL-1β treatment, respectively (Figures 4a,c). Because Cox-2 is a known downstream target of growth factor signalling pathways [12,13], we explored the possibility that activation of iFGFR1 also induces Cox-2 expression. HC-11/R1 cells were treated with AP20187 to activate iFGFR1 and Cox-2 expression was analysed by both quantitative RT-PCR and immunoblot analysis. As shown in Figure 4b, Cox-2 mRNA and protein expression were rapidly induced in the HC-11/R1 cells following iFGFR activation. Because Cox-2 is induced by both iFGFR1 and IL-1β in the HC-11/R1 mammary epithelial cells, we predicted that activation of both growth factor and cytokine-induced signalling pathways would result in a cooperative induction of Cox-2 expression. In agreement with this prediction, quantitative RT-PCR analysis revealed an additive increase in Cox-2 expression following activation of both iFGFR1 and IL-1β signalling pathways in comparison with either stimulation alone (Figure 4d). These results suggest that signalling pathways induced by growth factors and cytokines cooperate to promote increased levels of expression of inflammatory mediators. To determine whether iFGFR1-induced expression of Cox-2 is dependent on IL-1β, an IL-1β blocking antibody was added to the media at the time of AP20187 stimulation and Cox-2 expression was analysed by immunoblot analysis. We found that inhibition of IL-1β activity did not significantly affect Cox-2 expression levels (data not shown), suggesting that iFGFR1 induces Cox-2 expression in an IL-1β-independent manner and it is likely that IL-1β and iFGFR1 act through different signalling pathways to induce Cox-2 expression.

Because Cox-2 has been implicated in promoting breast cancer cell motility [29], we hypothesised that induction of Cox-2 was required for the synergistic effect of iFGFR1 and IL-1β on migration. To examine this hypothesis, HC-11/R1 cells were treated with the Cox-2 selective inhibitor, celecoxib. As shown in Figure 4e, addition of 25 μM celecoxib to the media resulted in a significant inhibition of migration induced by iFGFR1 activation. Furthermore, celecoxib also inhibited the migration induced by both iFGFR1 and IL-1β (Figure 4e), suggesting that Cox-2 is a critical mediator of migration induced by iFGFR1 activation and by iFGFR1/IL-1β co-stimulation.

### iFGFR1 activation in mammary epithelial cells induces expression of Cox-2 *in vivo*

**Figure 5:**
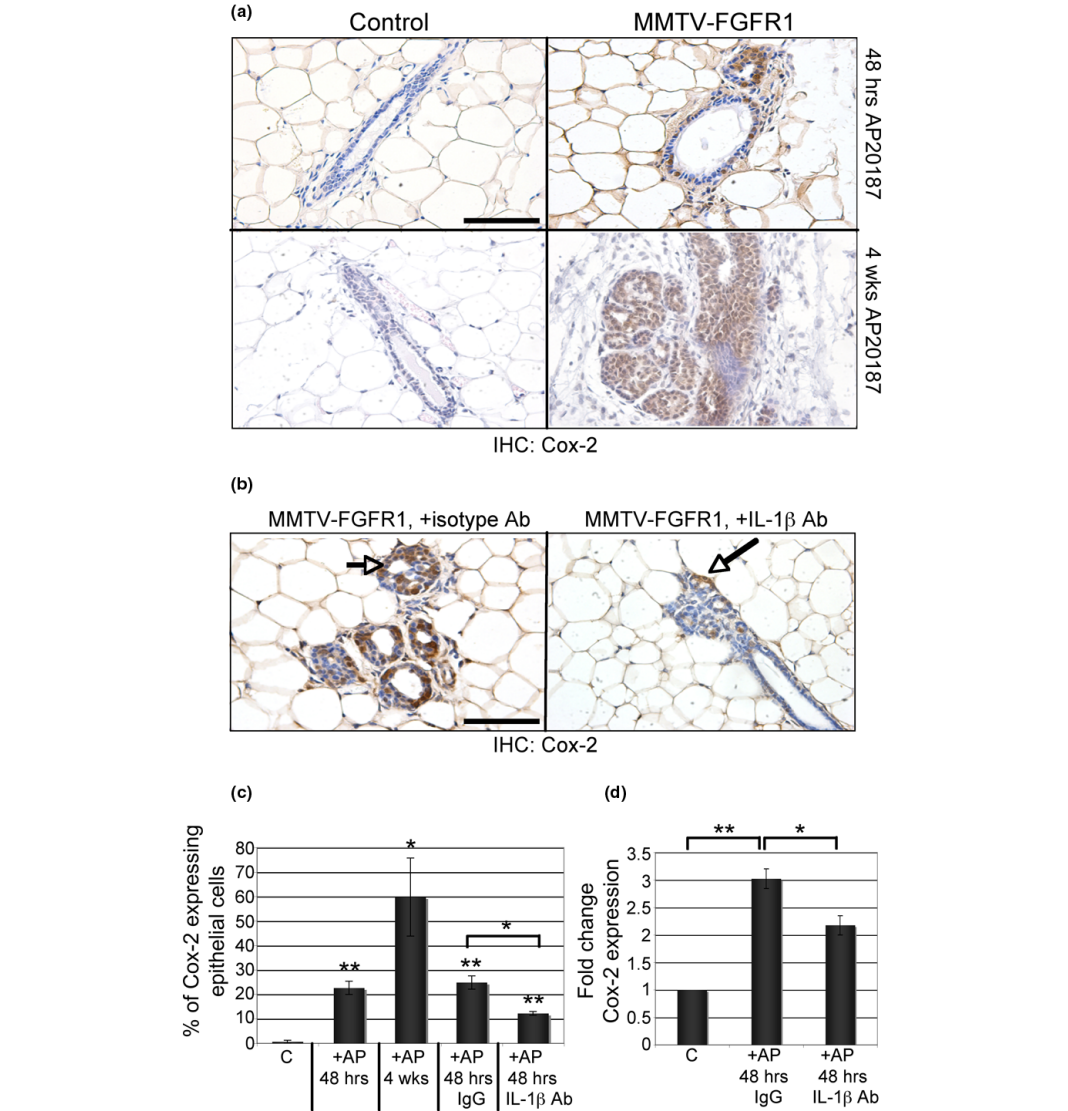
Activation of iFGFR1 induces Cox-2 expression *in vivo*, which is regulated in part by IL-1β activity. **(a) **Cyclooxygenase (Cox) 2 immunohistochemistry of mammary gland sections from either non-transgenic or transgenic mice treated with AP20187 for either 48 hours or 4 weeks. **(b) **Cox-2 immunohistochemistry of mammary gland sections from transgenic mice treated with either an isotype goat IgG or a neutralising IL-1β antibody in conjunction with AP20187 treatment. Magnification is 50 μM. The figure is representative of sections from three mice per treatment. **(c) **Percentage of epithelial cells expressing Cox-2 in panels a and b. Error bars represent standard error of the mean. * *P *< 0.05, ** *P *< 0.01. **(d) **Quantitative RT-PCR was used to analyse Cox-2 expression in mammary glands from non-transgenic and transgenic mice following treatment with AP20187 and either IgG or IL-1β blocking antibody. Cox-2 expression was normalised to cyclophilin expression. Error bars represent standard error of the mean. * *P *< 0.05, ** *P *< 0.01. Ab = antibody; IHC = immunohistochemistry; MMTV = mouse mammary tumour virus; NF = nuclear factor; iFGFR1 = inducible fibroblast growth factor receptor 1.

To explore the mechanisms by which IL-1β promotes the iFGFR1-induced mammary phenotype *in vivo*, we examined expression levels of Cox-2 following iFGFR1 activation using immunohistochemistry. As shown in Figure 5, increased expression of Cox-2 was observed in the mammary gland within 48 hours of iFGFR1 treatment (Figures 5a,c). Analysis of mammary glands following four weeks of treatment demonstrated sustained increased levels of Cox-2 associated with hyperplastic lesions (Figures 5a, c). To correlate the expression of Cox-2 with IL-1β activity, we examined Cox-2 expression in mammary gland sections from the mice treated with the IL-1β neutralising antibody. In comparison with mammary glands from mice treated with the IgG isotype control antibody, inhibition of IL-1β activity resulted in decreased Cox-2 expression in epithelial structures following 48 hours of treatment (Figures 5b, c). Interestingly, Cox-2 expression was not completely abolished and remained detectable in some cells (Figure 5c, arrow). Furthermore, analysis of Cox-2 mRNA expression analysed in the mammary glands by quantitative RT-PCR analysis demonstrated a decrease in Cox-2 gene expression in mammary glands from the mice treated with the IL-1β blocking antibody (Figure 5d). Consistent with the immunohistochemistry studies, Cox-2 gene expression was only partially reduced (Figure 5d). These results suggest that activation of iFGFR1 in mammary epithelial cells *in vivo *results in induction of Cox-2, which is mediated in part by IL-1β activity. However, inhibition of IL-1β activity did not completely abolish Cox-2 expression, suggesting that iFGFR1 may induce Cox-2 expression via alternate pathways as suggested by the *in vitro *studies.

### Cox-2 promotes the formation of iFGFR1-induced mammary lesions

**Figure 6:**
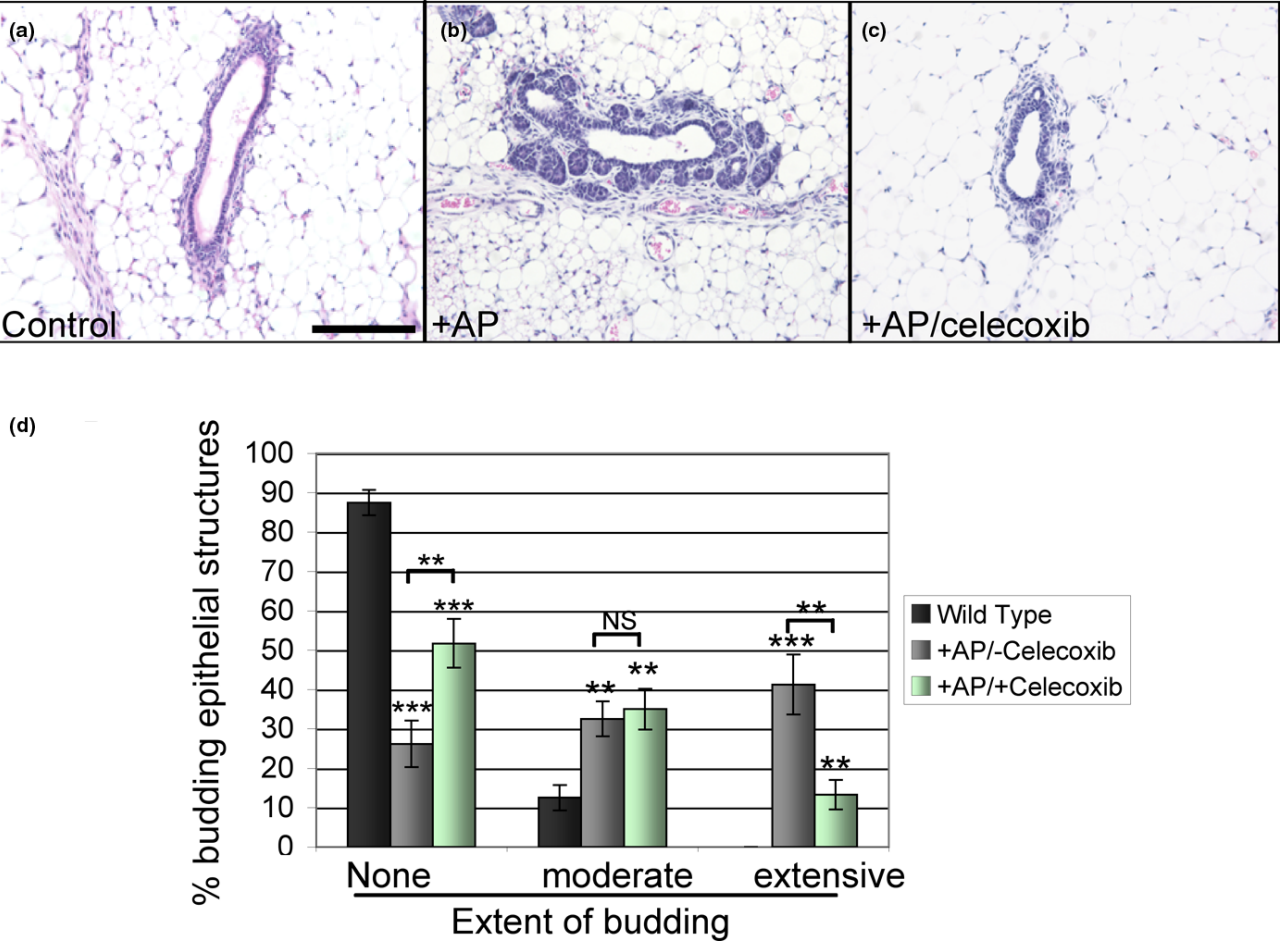
Effects of celecoxib on iFGFR1-induced mammary tumourigenesis. **(a to c) **H&E-stained sections from non-transgenic control mice and mouse mammary tumour virus (MMTV) inducible fibroblast growth factor receptor 1 (iFGFR1) transgenic mice following treatment of mice with AP20187 in the presence or absence of celecoxib showing example of representative budding structures observed in each group of mice. Magnification bar = 50 μM. **(d) **Extent of epithelial budding was quantified in sections from the mice described in panel a. Structures were classified as either containing no discernible budding structures (none), one to three lateral buds (moderate), or four or more budding structures (extensive) Error bars represent standard error of the mean. ** *P *< 0.01, *** *P *< 0.001. NS = not significant.

Although studies have demonstrated that Cox-2 promotes late-stage mammary tumourigenesis [30], the role of Cox-2 in the initial formation of early proliferative mammary lesions is unknown. Therefore, to examine the role of Cox-2 in this model, mice were provided with standard mouse chow supplemented with celecoxib for one-week prior to treatment with AP20187 to activate iFGFR1. Mice were then injected i.p. with AP20187 for 48 hours before mammary glands were removed for further analysis. Tissue samples were then stained with H&E for quantification of budding epithelial structures. As expected, mammary glands from mice treated with AP20187 in the absence of celecoxib exhibited increased extensive budding epithelial structures in comparison to mammary glands from non-transgenic mice treated with AP20187 (Figures 6a to 6d). Although celecoxib treatment did not completely eradicate the hyperplastic budding structures, there was a significant decrease in the percentage of extensive budding structures in mammary glands from mice given celecoxib-enhanced chow following AP20187 treatment (Figure 6d). Furthermore, the number of epithelial structures with no discernible budding was increased on treatment with celecoxib (Figure 6d), suggesting a delay in the formation of the iFGFR1-induced lateral budding phenotype. Notably, Cox-2 inhibition did not appear to inhibit formation of the hyperplastic lesions as well as IL-1β inhibition, as determined by observing whole mounts following celecoxib treatment (data not shown). This suggests that either IL-1β blocking antibodies are more efficient than celecoxib or that IL-1β can act through other downstream targets in the mammary gland. Overall, these data demonstrate that IL-1β and Cox-2 are involved in the formation of the iFGFR-induced hyperplastic phenotype and that targeting inflammatory mediators during early stages of mammary tumourigenesis may result in decreased formation of proliferative lesions.

## Discussion

Inflammation has been linked to the development of many types of cancer, including breast cancer [1,31]. Epidemiological studies have suggested that the use of non-steroidal anti-inflammatory drugs (NSAIDs) can decrease the relative risk of developing breast cancer [3-7,32,33]. These studies have led to the recent exploration of the use of Cox-2 inhibitors to treat breast cancer patients [32,34]. However, the origins of inflammation in breast cancer are still not well understood. Unlike other cancers in which chronic inflammation is associated with extrinsic factors such as bacterial or viral infection, breast cancer has not been linked to extrinsically induced inflammatory stimuli. Therefore, understanding the mechanisms by which inflammation might be induced in the breast will lead to important insights into developing anti-inflammatory strategies that may prevent the development and recurrence of invasive breast cancer.

We previously demonstrated that activation of iFGFR1 in mammary epithelial cells *in vivo *induced a rapid inflammatory response characterised by induction of inflammatory genes and recruitment of macrophages [14]. We have further used this model to explore the role of inflammation in promoting the formation of iFGFR1-induced hyperplastic lesions. For the current studies, we have focused on IL-1β, which is a potent proinflammatory cytokine that has been linked to breast cancer invasiveness and recurrence [8,35]. Studies of IL-1β in breast cancer have demonstrated that IL-1β expression is increased in 90% of oestrogen receptor-negative invasive breast carcinomas, and that it is localised to both tumour cells and stromal cells [9,36]. *In vitro *studies of breast cancer cells have demonstrated the ability of IL-1β to promote proliferation and migration [27,28]. However, the mechanisms by which IL-1β acts on tumour cells and/or cells within the stroma to promote breast cancer *in vivo *are not well understood.

Because our results clearly demonstrate increased IL-1β expression in the mammary gland following iFGFR1 activation, we were interested in identifying the cell types responsible for IL-1β production following iFGFR1 activation. Although activation of oncogenes has been shown to induce expression of IL-1β in tumour cells, we were unable to detect secreted IL-1β in the media of HC-11/R1 cells following iFGFR1 activation (data not shown). Furthermore, treatment of HC-11/R1 cells with an IL-1β blocking antibody at the time of AP20187 stimulation did not affect expression of Cox-2, suggesting that iFGFR1-induced expression of Cox-2 through an IL-1β independent pathway. However, co-culture of HC-11/R1 cells with either RAW 264.7 cells or bone marrow derived macrophages with HC-11/R1 cells resulted in a significant increase in IL-1β mRNA and protein secretion. Based on these studies, we propose that the majority of the IL-1β being produced *in vivo *is coming primarily from the macrophages, which are known to express high levels of IL-1β during inflammatory reactions. However, it is possible that IL-1β is also secreted by other cell types in the mammary gland, such as fibroblasts. Further studies are required to delineate the mechanisms by which IL-1β expression is induced in this model.

It is clear that iFGFR1 activation promotes an increase in IL-1β within the microenvironment, which can then act on the epithelial cells to promote tumourigenesis. Therefore, further studies were performed to examine the specific effects of exogenous IL-1β on epithelial cells to mimic the increased levels of IL-1β within the microenvironment. Recent studies have demonstrated that inflammatory cytokines have profound effects on mammary epithelial cell function [37]. For example, treatment of breast cancer cells with IL-1β has been reported to activate the NFκB pathway and induce both cell migration and proliferation [27,28,35]. However, the effects of IL-1β on normal mammary epithelial cells have not been described. Therefore, we initially evaluated the effects of IL-1β on NFκB activation, proliferation and migration. IL-1β alone activated NFκB, suggesting that the IL-1R signalling pathway is intact in mammary epithelial cells. However, treatment of cells with IL-1β did not promote either migration or proliferation of the HC-11/R1 cells. Interestingly, we found that activation of iFGFR1 in the presence of exogenous IL-1β resulted in increased migration compared with iFGFR1 activation alone. In contrast to the lack of effect of IL-1β on iFGFR1-induced proliferation of the HC-11/R1 cells, the IL-1β neutralising studies suggested that IL-1β promotes mammary epithelial cell proliferation *in vivo*. Therefore, it is important to consider that inflammatory mediators may have different effects on epithelial cells depending on the context of the microenvironment. In addition, it is possible that IL-1β affects mammary epithelial cell proliferation indirectly by acting on other cell types in the mammary gland. Further studies in which IL-1β signalling is impaired in specific cell types in the mammary gland would be required to elucidate these interactions.

Because Cox-2 is a downstream target of IL-1β, further studies focused on the ability of IL-1β to promote expression of Cox-2 in mammary epithelial cells. Cox-2 is expressed in human breast tumours and has been found in both early-stage atypical hyperplasias and invasive cancers [38]. However, recent studies have suggested that in contrast to previous studies, Cox-2 expression is decreased in invasive breast cancers [34,39,40]. Therefore, although the use of Cox-2 inhibitors in late-stage breast cancer may be limited, epidemiological studies suggest that Cox-2 inhibition may be relevant for breast cancer prevention. Interestingly, our results suggest that induction of Cox-2 expression alone is insufficient to drive tumourigenic alterations of normal epithelial cells such as migration and proliferation, because treatment of cells with IL-1β alone resulted in increased Cox-2 but did not promote these phenotypes. However, in combination with a single oncogenic stimulus, iFGFR1 activation, Cox-2 was able to participate in the acquisition of cell motility. These data suggest that the functions of Cox-2 in breast cancer are likely to be dependent on the stage of cancer and the context of the microenvironment rather than the levels of protein expression.

Cox-2-selective inhibitors have previously been shown to inhibit mammary tumour formation in mouse mammary tumour models [30]. However, due to the stochastic nature of these tumour models, it is difficult to determine the effects of Cox-2 inhibition during the initiating events in tumourigenesis. Because there is substantial interest in the ability of anti-inflammatory drugs to reduce the risk of developing breast cancer, inducible models of mammary tumourigenesis provide a unique opportunity to study the contributions of inflammation to breast tumourigenesis. Our results from the Cox-2 inhibition *in vivo *studies demonstrate that inhibition of Cox-2 prior to activating the initial oncogenic stimulus may be sufficient to delay the formation of early-stage lesions in the breast. Interestingly, gross comparison of the mammary glands using whole mounts suggested that Cox-2 inhibition did not appear to be as effective as IL-1β inhibition. Therefore, comparisons between different methods of inhibiting IL-1β and Cox-2 in this model may provide insights into which of these inflammatory mediators might represent a more effective anti-inflammatory target. Finally, further studies of this model will provide important insights into the role of inflammation in promoting breast tumour formation, which may be particularly relevant to subsets of patients that have high levels of inflammatory mediators associated with pre-invasive lesions in the breast.

## Conclusions

Studies of growth factor signalling pathways in breast cancer have produced a wealth of information regarding the effects of these pathways on tumour formation and progression. However, it is becoming more apparent that tumours are the result of a complex interplay bet ween intracellular signalling pathways and extracellular stimuli. Although several growth factor receptors, such as epidermal growth factor receptor, ErbB2 and FGFR1, have been studied and implicated in breast cancer, we are only beginning to understand how these growth factor pathways interact with other autocrine or paracrine factors to promote tumourigenesis. Although inflammatory cytokines have been implicated in breast cancer, it is clear that these cytokines alone are not sufficient to cause tumour formation. In fact, it is well accepted that cancer does not develop due to a single genetic alteration, but instead requires the contribution of multiple factors, both within the cell and in the microenvironment. Understanding how these various factors cooperate within epithelial and tumour cells to promote breast cancer initiation and formation will lead to the development of more effective combinatorial therapies designed to target multiple pathways within both the tumour cells and the supporting stromal cells.

## Abbreviations

Cox-2: cyclooxygenase 2; ELISA: enzyme linked immunosorbent assay; FGFR1: fibroblast growth factor receptor 1; GAPDH: glyceraldehyde 3-phosphate dehydrogenase; H&E: haematoxylin and eosin; iFGFR1: inducible fibroblast growth factor receptor 1; Ig: immunoglobulin; IL: interleukin; IL-1R: interleukin-1 receptor; i.p.: intraperitoneally; MMTV: mouse mammary tumour virus; NF: nuclear factor; NSAID: non-steroidal anti-inflammatory drug; pH3: phospho-histone H3; rm: recombinant murine; RT-PCR: reverse transcription polymerase chain reaction.

## Competing interests

The authors declare that they have no competing interests.

## Authors' contributions

JRR performed cell culture studies and the *in vivo *celecoxib studies, analysed the data and prepared results and contributed to drafting of the manuscript. RPL participated in the cell culture studies and performed quantitative RT-PCR analysis. MKH maintained the animal colony including breeding and genotyping, and performed cell culture studies. KLS conceived of the study, directed the research, performed the IL-1β blocking studies and drafted the manuscript.

## Supplementary Material

Additional file 1 A JPG file containing images of whole mount analysis of mammary glands following inducible fibroblast growth factor receptor 1 activation and IL-1β inhibition. Mammary glands from non-transgenic, non-treated mice (control) and transgenic mice treated with AP20187, IgG isotype control or IL-1β blocking antibody were analysed by whole mount analysis. Click here for file
