# Supplementary figures and images for: Interleukin-1beta and fibroblast growth factor receptor 1 cooperate to induce cyclooxygenase-2 during early mammary tumourigenesis
Source: Breast Cancer Res. 2009 Apr 24;11(2):R21. doi: 10.1186/bcr2246 (PMC2688950; doi:10.1186/bcr2246)

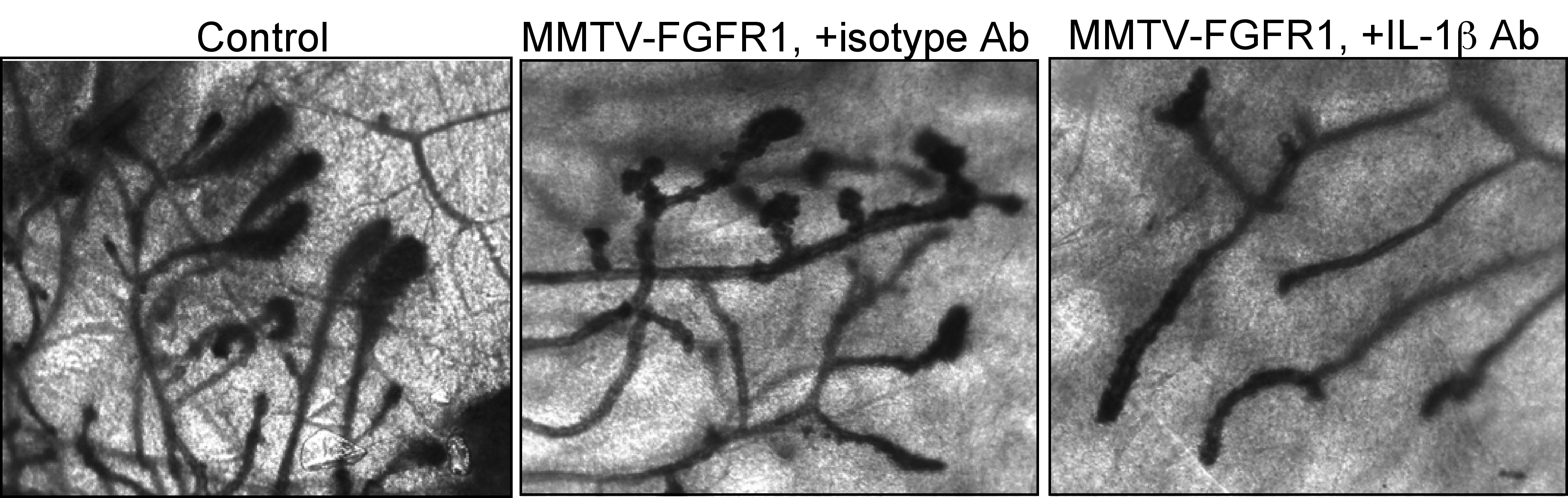

Supplement: Additional file 1 — A JPG file containing images of whole mount analysis of mammary glands following inducible fibroblast growth factor receptor 1 activation and IL-1β inhibition. Mammary glands from non-transgenic, non-treated mice (control) and transgenic mice treated with AP20187, IgG isotype control or IL-1β blocking antibody were analysed by whole mount analysis. [file bcr2246-S1.jpeg]
